# Human Dignity as a Criterion for Science Policy

**DOI:** 10.1371/journal.pmed.0020244

**Published:** 2005-08-02

**Authors:** Timothy Caulfield, Audrey Chapman

## Abstract

A lack of clarity in defining human dignity, argue Caulfield and Chapman, has the potential to hurt policymaking in science and medicine.

Increasingly, the question of whether some forms of research, such as human cloning, infringe notions of human dignity has been proposed as a primary justification for the development of science policy. This is understandable. A commitment to human dignity is a widely shared value and the foundation for our understanding of human rights. The preamble to the Universal Declaration of Human Rights, adopted by the United Nations General Assembly in 1948, states that “recognition of the inherent dignity and of the equal and inalienable rights of all members of the human family is the foundation of freedom, justice, and peace in the world” [1].

However, in the context of emerging scientific advances, such as cloning technology and stem cell research, exactly how to judge whether human dignity is infringed upon or degraded is rarely explained. As an example, a 2002 report by the President's Council on Bioethics is titled “Human Cloning and Human Dignity: An Ethical Inquiry,” but it fails to conceptualize human dignity or address the specific ways in which human cloning may impinge on human dignity [2].

This lack of clarity has the potential to hurt policymaking and, in the long run, degrade the possible substantive value of the principle of human dignity. The dilemma is that human dignity is a poorly conceptualized and vague concept. Further complicating matters, in a pluralistic society various groups and communities bring a diversity of worldviews, religious values, and cultural understandings that inform and shape their use of the concept of human dignity.

## The Concept of Dignity

There are numerous examples of policies that cite human dignity as a standard for dealing with controversial science issues. The UN Educational, Scientific, and Cultural Organization's “Universal Declaration on the Human Genome and Human Rights” recommends a ban on “practices which are contrary to human dignity, such as reproductive cloning” [3]. A 2003 World Health Organization report suggests that genetic databases create the need to balance “human dignity and human rights as against public health, scientific progress and commercial interests in a free market” [4]. Both Japan's 2001 stem cell research guidelines and Canada's recent legislation covering research involving human reproductive material claim the protection of human dignity as a primary objective of the regulatory regimes [5,6]. And, of course, the concept of human dignity permeates research ethics policy (e.g., the Helsinki Declaration). Canada's primary research ethics document, the “Tri-Council Policy Statement,” declares that the “cardinal principle of modern research ethics, as discussed above, is respect for human dignity” [7].

In these documents, the concept of human dignity is often used in the conventional legal and ethical manner to emphasize the right of individuals to make autonomous choices. This is most apparent in the context of research ethics documents and informed-consent policies. This conception treats human dignity as a means of empowerment. Some scholars have gone so far as to suggest that this is the only appropriate normative use of the idea of dignity [8].

Despite such claims, an alternative conception, dignity as a means of constraint, is increasingly common in the realm of science policy. Citations of human dignity in science policy discussions usually come in the context of concerns that some activities, such as human cloning and the commodification of human tissue, infringe some basic understanding of dignity [9]. Costa Rica's recent proposal to the UN for an international treaty banning cloning stands as a good example of this trend. The Costa Rican draft convention sought to “ensure respect for the dignity and basic rights of the human being” in the face of the “threat posed by experiments in the cloning of human beings” [10]. Likewise, in the area of stem cell research, opponents refer to the dignity implications as a rationale for limiting research on human embryos. In Europe, it is an underpinning of the “ordre public” (public policy) restriction of patent law, which has been used to deny patents on cloning technologies and human embryonic stem cells [11]. [Fig pmed-0020244-g001]

**Figure pmed-0020244-g001:**
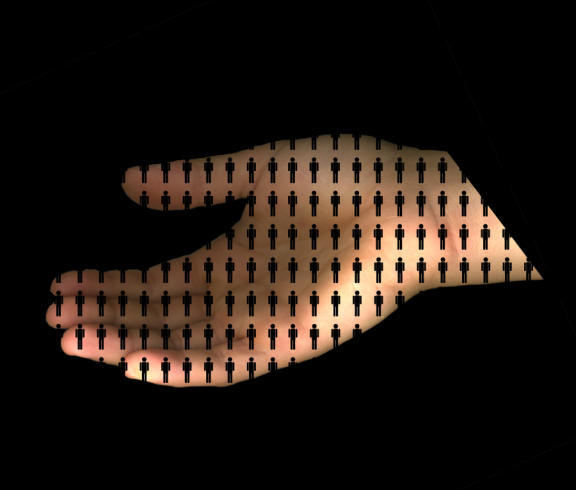
Some policymakers suggest that human cloning would infringe on dignity, but they rarely explain how to judge such infringement (Illustration: Margaret Shear, Public Library of Science)

When used in this manner, dignity is meant to reflect a broad social or moral position that a particular type of activity is contrary to public morality or the collective good. It is not used as a source of individual rights, but as a justification for a policy response—usually a policy that is intended to curtail a given activity.

While most would agree that human dignity is closely tied to the idea of the inherent worth of humans, policy documents and legal instruments rarely provide an explicit definition of dignity or how human worth might be degraded by a given technology or scientific activity. “[Dignity's] intrinsic meaning has been left to intuitive understanding, conditional, in large measure, by cultural factors” [12]. As such, its meaning will be very different depending on the values and background that an individual or community brings to the deliberations.

## Problems with “Dignity”

Why is this lack of clarity problematic? At worst, the concept of dignity appears to often be used as mere rhetorical dressing, adding little more to the policy debate than the weight or cachet of the concept. In such circumstances, dignity conveys a sense of general social unease, but with little explanation of how, exactly, human dignity is threatened. At times, reference to human dignity seems to have emerged, inappropriately, as a politically palatable articulation of the “yuck factor”—a way of “registering our concern” about activities that seem to threaten “those parts of the human condition that are familiar and reassuringly ‘human,’” without the detailed explanation of why and how the activities are troubling [13].

The use of dignity as a policy justification can silence open debate, and may serve to blur an understanding of the real policy concerns behind a given technological innovation or scientific development. Moreover, without a clearer conception of human dignity and its requirements, it is not possible to evaluate technological innovation or scientific developments in the service of protecting human dignity.

In addition, because human dignity is viewed as a foundational concept, its use may imply a degree of social consensus that simply does not exist. If something is said to infringe human dignity, one would expect a degree of agreement that this is so. However, modern societies are often pluralistic, and in pluralistic societies, consensus is often difficult to obtain, whether about human dignity or other complex social and ethical issues introduced by scientific innovations [14]. There is not even agreement about the foundation of human dignity—whether it is faith-based or secular—let alone what human dignity entails. In the debate on stem cell research, for example, not all agree on the moral status of the embryo and, as such, they cannot reach agreement on the degree to which embryonic stem cell research challenges human dignity.

As a result, the use of dignity will not necessarily represent a broadly accepted social value, but, instead, it may express a particular worldview—a worldview that may not even reflect the majority opinion. In the context of legal instruments and policy documents, the use and content of human dignity may amount to a “political compromise informed by cultural, political, constitutional and other conditions, all of which can then evolve and change” [15]. In more extreme circumstances, it could involve “intolerant voices (whether of the majority or of an influential minority) expressing negative attitudes about certain practices, which attitudes are then translated into restrictions ostensibly in the interest of respect for human dignity” [9].

## The Value of Human Dignity to Science Policy

For the reasons cited above, we need to be cautious not to simply accept the concept of human dignity as a primary or sole justification for science policy. As noted by Hayry, “ethical controversies cannot be settled simply by stating that this or that solution respects or violates human dignity” [16].

Nevertheless, despite the concerns that have been articulated about the recent constraining and vague use of dignity in science policy, we believe that it remains a potentially useful normative concept. Indeed, the fact that the concept of human dignity “anchors different worldviews” may, paradoxically, be its greatest policy value. Most, if not all, agree that human dignity is tied to notions of human worth [15]. As such, a pronouncement that something infringes human dignity should be viewed as an opportunity to debate the values at play and the cultural underpinnings of the concern. Hayry concludes thus: “People can debate the merits of different notions of dignity, and they can argue the relative significance of opposing concepts in ethical disputes. Although it is probably true that dignity should never be used as a mere means, the concept of dignity could in this way be used as a means to further understanding between people and cultures” [16].

The key to its constructive use, however, is that dignity be used as a facilitator of policy debate, instead of a “door closer.” This means the concept of human dignity should not be used as a slogan or as part of a mere assertion of harm. It also means that when human dignity is used as a grounding for science policy—be it as a justification for empowerment or technological constraint—as much specificity as possible should be provided. For example, how does cloning technology challenge the intrinsic worth of humans? Is it the mere replication of nucleic DNA or something else? Do the dignity concerns about stem cell research go beyond disputes about the moral status of the embryo?

We believe that the close connection to the notion of human worth differentiates it from mere “intuitive” responses to a technology and, as such, should force an articulation of the way in which various communities view the foundations of human nature and worth. In a pluralistic society, this may require exploring the various approaches to dignity in different cultures and communities.

Finally, communities will need to consider how our community-specific conceptions of dignity relate to broader regulatory policy. It may be difficult to develop a law or policy that can respect the diverse conceptions of human dignity, as highlighted by the divisive nature of the stem cell debate. But by using the concept of human dignity as an avenue for exploring differing philosophical approaches, we promote transparency, encourage dialogue, and help to avoid the simplistic application of dignity in science policy.

## Conclusion

The issues being raised about the impact of scientific discoveries and new technologies on human dignity make it imperative to gain greater understanding about the meanings and requirements of this important but elusive concept. Otherwise, references to dignity will likely be ineffectual and potentially even a source of social division. Moreover, the one goal that all groups may agree upon, the importance of protecting human dignity, will never be achieved.
